# Exome, transcriptome and miRNA analysis don’t reveal any molecular markers of TKI efficacy in primary CML patients

**DOI:** 10.1186/s12920-019-0481-z

**Published:** 2019-03-13

**Authors:** Alexander V. Lavrov, Ekaterina Yu. Chelysheva, Elmira P. Adilgereeva, Oleg A. Shukhov, Svetlana A. Smirnikhina, Konstantin S. Kochergin-Nikitsky, Valentina D. Yakushina, Grigory A. Tsaur, Sergey V. Mordanov, Anna G. Turkina, Sergey I. Kutsev

**Affiliations:** 1 Laboratory of Mutagenesis, Federal State Budgetary Institution, Research Centre for Medical Genetics, Moskvorechie str., 1, Moscow, Russian Federation 115522; 2 Scientific and Advisory Department of Chemotherapy of Myeloproliferative disorders, Federal State-Funded Institution National Research Center for Hematology of the Ministry of Healthcare of the Russian Federation, Novy Zykovki proezd, 4, Moscow, Russian Federation 125167; 3 Department of Molecular and Cellular Genetics, State Budgetary Educational Institution of Higher Professional Education “Russian National Research Medical University named after N.I. Pirogov” of Ministry of Health of the Russian Federation, Ostrovityanova str., 1, Moscow, Russian Federation 117997; 4 Regional Children Hospital 1, S. Deryabinoy str., 32, Ekaterinburg, Russian Federation 620149; 5 Research Institute of Medical Cell Technologies, Soboleva str., 25, Ekaterinburg, Russian Federation 620905; 6 Federal State Budgetary Educational Institution of Higher Education, Urals State Medical University of the Ministry of Healthcare of the Russian Federation, Repina str., 3, Ekaterinburg, Russian Federation 620028; 7 Laboratory of Medical Genetics, The Rostov State Medical University, Nahichevansky av., 29, Rostov-on-Don, Russian Federation 344022

**Keywords:** Chronic myeloid leukemia, TKI efficacy, Exome, Transcriptome, miRNA analysis

## Abstract

**Background:**

Approximately 5–20% of chronic myeloid leukemia (CML) patients demonstrate primary resistance or intolerance to imatinib. None of the existing predictive scores gives a good prognosis of TKI efficacy. Gene polymorphisms, expression and microRNAs are known to be involved in the pathogenesis of TKI resistance in CML. The aim of our study is to find new molecular markers of TKI therapy efficacy in CML patients.

**Methods:**

Newly diagnosed patients with Ph+ CML in chronic phase were included in this study. Optimal and non-optimal responses to TKI were estimated according to ELN 2013 recommendation. We performed genotyping of selected polymorphisms in 62 blood samples of CML patients, expression profiling of 33 RNA samples extracted from blood and miRNA profiling of 800 miRNA in 12 blood samples of CML patients.

**Results:**

The frequencies of genotypes at the studied loci did not differ between groups of patients with an optimal and non-optimal response to TKI therapy. Analysis of the expression of 34,681 genes revealed 26 differently expressed genes (*p* < 0.05) in groups of patients with different TKI responses, but differences were very small and were not confirmed by qPCR. Finally, we did not find difference in miRNA expression between the groups.

**Conclusions:**

Using modern high-throughput methods such as whole-exome sequencing, transcriptome and miRNA analysis, we could not find reliable molecular markers for early prediction of TKI efficiency in Ph+ CML patients.

**Electronic supplementary material:**

The online version of this article (10.1186/s12920-019-0481-z) contains supplementary material, which is available to authorized users.

## Introduction

Chronic myeloid leukemia (CML) is a clonal myeloproliferative disorder characterized by the presence of [9;22] translocation and *BCR/ABL* fusion gene with high tyrosine kinase activity which activates MAPK pathway, cell proliferation, blocks apoptosis and leads to genome instability resulting in further development of the disease. Imatinib, BCR/ABL tyrosine kinase inhibitor (TKI) is the standard therapy in CML-Ph+ patients since its FDA approval in 2001. Patient’s survival improved from 7.5 years after diagnosis before imatinib era to 17.5 years now-a-days [1].

Despite high efficacy of imatinib the problem of primary resistance persists. Based on the recent report about 21% of CML patients are switched to another TKI because of resistance or intolerance [2]. According to other authors approximately 20 to 30% of patients develop resistance to imatinib [3].

At the same time hematologists have limited instruments to determine which patients will have primary resistance to imatinib and may benefit from other treatment regimens or use of newly developed TKIs as the 1st line therapy. Sokal and Hasford scores were developed in pre-imatinib era and now poorly predict the outcomes of the TKI therapy while EUTOS score gives more reliable prediction [4]. However in some studies it was estimated that all three scoring systems didn’t work correctly to predict complete cytogenetic response and survival with imatinib treatment, especially in non-European populations [5, 6]. Currently, the main trend to predict the better outcome is to use the rule “deeper and earlier response” [7], but this approach allows only late prediction after starting the therapy.

Different genetic factors are known to be associated with primary imatinib resistance [8, 9]. A variety of polymorphisms and mutations in genes associated with TKI resistance was reported [10–12]. Recently we have performed whole-exome sequencing in primary CML patients before TKI administration and revealed five genetic variants typical for optimal responders (rs11579366 in *ANKRD35*, rs1990236 in *DNAH9*, rs176037 in *MAGEC1*, rs10653661 in *TOX3*, rs3803264 in *THSD1*) and two – for non-optimal responders (rs3099950 in *MORN2*, rs9471966 in *PTCRA*) [13].

Among other factors gene expression differences are of a particular interest since expression profiling was demonstrated to be a valuable prognostic tool in many types of cancer. It was previously shown that expression levels of several genes differ between groups with different responses to TKI therapy [14, 15]. Frank et al. demonstrated that *COCH*, *ANAPC5* and *TPSAB1/B2* were overexpressed in imatinib non-responders while *VNN1* and *RPH3A* were down-regulated. The magnitude of differences was dramatically low – fold change varied from 0.547 to 1.487 and was confirmed by qRT-PCR for only two genes. However 128-gene expression signature was successfully used to correctly classify the subset of test samples [14].

MircoRNAs (miRNAs) are small (18–25 nucleotides in length) non-coding RNAs which regulate gene expression by translational repression or mRNA cleavage [16]. Previously obtained data shows that miRNAs are involved in CML pathogenesis: some miRNAs are up-regulated and some are down-regulated in the peripheral blood of CML patients [17–20]. Moreover, there is data supporting the idea of different expression levels of miRNAs in CML patients with good and poor response to TKI therapy. San José-Enériz et al. [21] performed analysis of expression profiles of 250 miRNAs in bone marrow mononuclear cells from patients with Ph+ CML at diagnoses and showed that 19 miRNAs were differentially expressed in resistant and responder samples. Similar study was performed in peripheral blood samples by microarray analysis in two groups of patients – with response and resistance to TKI. Authors identified 70 differently expressed miRNAs between these groups [22]. In both studies cluster unsupervised analysis of obtained expression levels of miRNAs was able to distinguish clearly both groups. It was also shown that miR-30 reduces *BCR/ABL* mRNA and protein levels by binding directly to the *ABL* 3′UTR and increases sensitivity of BCR/ABL-positive cells to imatinib. CML patients expressing low levels of miR-30 were less sensitive to imatinib [23]. High expression of miR-424 suppressed proliferation and induced apoptosis of K562 cells thereby increased sensitivity to imatinib treatment [24]. In another work it was shown that miR-26a, miR-29c, miR-130b and miR-146a were down-regulated in imatinib resistant patients in comparison to responders [25].

Despite the variety of the approaches and findings at DNA and RNA levels none of the potential markers were validated or implemented in clinical practice. The aim of our study is to find new molecular markers of TKI therapy efficacy in CML patients.

## Materials and methods

### Patients

Newly diagnosed patients with Ph+ CML in chronic phase were included in this study. Patients were recruited in the National Research Center for Hematology (Moscow), Regional Children Hospital #1 (Ekaterinburg), Research Institute of Medical Cell Technologies (Ekaterinburg) and Rostov State Medical University (Rostov-on-Don). The study was performed according to the provisions of the Declaration of Helsinki. Informed consent was obtained from all patients included in this study. European LeukemiaNet (ELN) Recommendations for Management of CML [26] were followed to form criteria of

- optimal response at 6 month: BCR-ABL^IS^ < 1% and/or 0% Ph+ metaphases out of at least 20 or more bone marrow cells;
- non-optimal response: BCR-ABL^IS^ 1–10% and/or > 1% Ph+ metaphases out of at least 20 or more bone marrow cells at 6 months.

### Genotyping of CML patients

Direct DNA Sanger sequencing of PCR products was performed as described previously [13]. Briefly, DNA from peripheral blood samples was obtained from CML patients before TKI therapy. Sequencing was performed using ABI Prism 3130XL genetic analyzer (Applied Biosystems, Foster City, CA) with either forward or reverse primer.

### Transcriptome and Bioinformatic analysis

Blood samples were collected in Paxgene tubes (Becton, Dickinson and Company, USA) at diagnosis before initiating TKI therapy. RNA was extracted using PAXgene Blood RNA Kit (Qiagen, Germany) according to the manufacture’s protocol. Expression profiling was performed using HumanHT Expression BeadChip (Illumina). Data analysis was performed in GenomeStudio_GX_Module (Illumina) and Partek Genomics Suite (Partek).

### miRNA and Bioinformatic analysis

Total RNA was extracted from blood samples and lymphocytes cell cultures obtained before beginning the therapy at the time of diagnosis CML. RNA from Paxgene tubes (Becton, Dickinson and Company, USA) was extracted using PAXgene Blood RNA Kit (Qiagen, Germany) according to the manufacture’s protocol; RNA from cell cultures was extracted by standard phenol-chloroform method. miRNA profiling of 800 miRNA was done with nCounter miRNA Expression Assay (Nanostring Technologies) according to the manufacturer’s protocol. Data analysis was performed in nSolver (Nanostring Technologies).

### Real-time reverse transcriptase-polymerase chain reaction

cDNA synthesis was performed using M-MuLV (SibEnzyme, Russia) with random hexa-primers. For validation of transcriptome findings cDNA (3 μl) was added to the reaction mixture (25 μl) containing 300 nM primers, 200 μM deoxynucleotide triphosphate, 1× working solution of SYBR Green I for *ACTB* and *B2M* genes (Invitrogene, USA), or 0.3 μM TaqMan probe for *UBA52*, *ATG7*, *PRR13* and *DAZAP2* genes and 0.04 U Taq-polymerase. Real-time PCR was performed on a CFX96 system (Bio-Rad, USA). The reaction protocol was as follows: 5 min at 95°С and 40 cycles (10 s at 95 °C, 10 s at 65 °C, 10 s at 70 °C). For *DAZAP2* gene protocol was as follows: 3 min at 95°С and 40 cycles (10 s at 95 °C, 10 s at 60 °C, 20 s at 70 °C). List of primers is presented in (Additional file 1: Table S1). The relative amount of mRNA *UBA52*, *ATG7*, *PRR13* and *DAZAP2* was calculated by standardized expression (ΔΔC (t)) for gene *ACTB*. The calculations were performed using CFX96 software (Bio-Rad, USA).

### Statistical analysis

Statistical calculations were performed using STATISTICA 64 ver.13 (Dell Inc., USA), MS Office Excel, and SPSS Statistics 21.0. Chi-square tests were used to process the obtained genotyping data. A significance level of 0.05 was set and Bonferroni correction for multiple comparisons was applied. Mann-Whitney U test for the two independent groups was used for expression data analysis, patients’ characteristics and comparison of numbers of obtained genetic variants. Haplotypes analysis was performed using R Haplo.stats [27]. Analysis of variance (ANOVA) and pathway enrichment analysis were performed in GenomeStudio_GX_Module (Illumina) and Partek Genomics Suite (Partek).

## Results

Whole exome sequencing (WES) was used to find new potential markers of TKI efficacy [Lavrov AV, 2016]. With the aim of finding new molecular markers of failure of the TKI therapy we performed validation of the WES findings, transcriptome and miRNA analyses.

### Prognostic role of the polymorphisms in cancer-associated genes

Seven variants were found to associate with the TKI efficacy in a small group of CML patients: rs11579366 (in *ANKRD35* gene), rs1990236 (*DNAH9*), rs176037 (*MAGEC1*), rs10653661 (*TOX3*), rs3803264 (*THSD1*), rs3099950 (*MORN2*), and rs9471966 (*PTCRA*). To validate these possible prognostic markers 62 patients with Ph+ CML in chronic phase were enrolled in the study. Median age was 46 years (range 19–71). Female:male ratio was 1:1.3. Fifty-nine patients received first-line therapy and three – second-line due to intolerance to imatinib or sub-optimal response at 3 months. According to ELN 2013 recommendations [26] at 6 months of TKI treatment all patients were divided into two groups – with optimal (*n* = 32) and non-optimal (*n* = 30) response to targeted therapy. Description of genotyped patients is available in Table 1.

**Table 1:** Characteristics of the genotyped patients

	Patients with optimal response at 6 months (*n* = 32)	Patients with non-optimal response at 6 months (*n* = 30)	*p*-value
Age, years, median (range)	39 (19–66)	52 (21–71)	0.0768
Gender			0.5940
Female, n (%)	15 (55.6)	12 (44.4)	
Male, n (%)	17 (48.6)	18 (51.4)	
Sokal group			
Low, n (%)	21 (65.6)	4 (13.3)*	
Intermediate, n (%)	6 (18.8)	1 (3.3)*	
High, n (%)	5 (15.6)	2 (6.7)*	
TKI therapy			
First line, n (%)	29 (90.6)	30 (100)	
Imatinib 400 mg, n (%)	25 (78.1)	28 (93.3)	
Imatinib 600 mg, n (%)		2 (6.7)	
Nilotinib 400–600 mg, n (%)	3 (9.4)		
Dasatinib, 100 mg, n (%)	1 (3.1)		
Second line, n (%)	3 (9.4)	0 (0)	
Nilotinib 400–600 mg, n (%)	1 (3.1)**		
Dasatinib, 100 mg, n (%)	2 (6.3)**		

*Sokal score data for 23 patients were not available

**patients were switched to the second line therapy due to imatinib intolerance or sub-optimal response at 3 months

*TKI* tyrosine kinase inhibitor

The results of the genotyping of all the patients are summarized in Table 2. The frequencies of genotypes at the studied loci did not differ between groups of patients with an optimal and non-optimal response to TKI therapy.

**Table 2:** Rates of genotypes of analyzed genes in groups of patients with optimal and non-optimal response to TKI therapy

Gene and reference SNP	Reference allele	Variant allele		Patients with optimal response at 6 months (*n* = 32)			Patients with non-optimal response at 6 months (*n* = 30)			*р*-value
*ANKRD35* rs11579366	G	C	genotype	G/G	G/C	C/C	G/G	G/C	C/C	0,16
			rate, %	9	50	41	29	39	32	
*DNAH9* rs1990236	G	A	genotype	G/G	G/A	A/A	G/G	G/A	A/A	0,33
			rate, %	72	22	6	57	39	4	
*MAGEC1* rs176037	C	T	genotype	C/C	C/T	T/T	C/C	C/T	T/T	0,68
			rate, %	53	20	27	41	32	27	
*TOX3* rs10653661	A	ATTTCT	genotype	A/A	A/ ATTTCT	ATTTCT/ ATTTCT	A/A	A/ ATTTCT	ATTTCT/ ATTTCT	0,87
			rate, %	22	56	22	23	50	27	
*THSD1* rs3803264	G	A	genotype	G/G	G/A	A/A	G/G	G/A	A/A	0,38
			rate, %	6	44	50	12	27	61	
*MORN2* rs3099950	G	A	genotype	G/G	G/A	A/A	G/G	G/A	A/A	0,48
			rate, %	69	31	0	77	23	0	
*PTCRA* rs9471966	G	A	genotype	G/G	G/A	A/A	G/G	G/A	A/A	0,88
			rate, %	56	38	6	50	43	7	

*SNP* single nucleotide polymorphism

Since genotype rates for individual genes did not demonstrate significant differences between groups of patients with optimal and non-optimal response to TKI, we analyzed whether there is an association of response to therapy with haplotypes (different combinations of alleles in selected genes). There was no reliable association with the therapy response of any of the haplotypes presented in patients (*p* = 0.94). Correction by gender and age also did not change the result.

### Transcriptome analysis

Thirty-three Ph+ CML patients with the follow-up period more than 6 months were treated in National Research Center for Hematology (Moscow). Most of the patients demonstrated good response to the TKI therapy. However, five didn’t have optimal response. All of them had > 10% Ph+ cells at 6 months (according to ELN2013 recommendations non-optimal response). We also chose 7 patients with optimal response for the comparison. Patient ages ranged from 20 to 77 years, with a median of 37 years. All 12 patients were treated by TKIs: 10 patients treated by imatinib, 2 patients by nilotinib. Detailed characteristics of these patients are available in Table 3.

**Table 3:** Characteristics of patients for transcriptome study

Patient	Gender	Age, years	Sokal score	TKI first line, dosage	Response to TKI
1	Male	36	Intermediate risk	Imatinib, 400 mg	Optimal response
2	Female	27	High risk	Imatinib, 600 mg	Optimal response
3	Female	57	Low risk	Imatinib, 400 mg	Optimal response
4	Female	55	Low risk	Imatinib, 400 mg	Optimal response
5	Female	68	Intermediate risk	Imatinib, 400 mg	Non-optimal response
6	Male	77	Low risk	Imatinib, 400 mg	Optimal response
7	Male	34	Intermediate risk	Imatinib, 400 mg	Non-optimal response
8	Male	20	Low risk	Nilotinib, 600 mg	Optimal response
9	Female	30	Low risk	Imatinib, 400 mg	Non-optimal response
10	Male	30	High risk	Nilotinib, 800 mg	Non-optimal response
11	Female	63	Intermediate risk	Imatinib, 400 mg	Non-optimal response
12	Female	38	Low risk	Imatinib, 400 mg	Optimal response

*TKI* tyrosine kinase inhibitor

ANOVA analysis of the expression of 34,681 genes revealed 26 differently expressed genes (*p* < 0.05) in patients with optimal and non-optimal responses (Table 4). Ten of these genes (*DAZAP2, UBA52, PRR13, ATG7, LOC387820, PAK1, RAB11A, EMP3, GSTM1, GSTM2*) are associated with other cancers and another four (*RSAD2, MAP3K11, COMMD1, TNFRSF1A*) are the key members of regulatory pathways including NF-kappa-B, MAPK, JNK and other thought to be disbalanced in many cancers (see Table 4).

**Table 4:** Differently expressed genes involved in cancers or regulatory pathways in optimal and non-optimal responders

Gene	Localization	Gene description	Coefficient	RT-PCR, x
*DAZAP2*	12q12	DAZ-associated protein 2	−2.14	−1.16
*UBA52*	19p13.1-p12	Ubiquitin A-52 residue ribosomal protein fusion product 1	−1.86	−1.47
*PRR13*	12q12	Proline rich 13	−1.58	−1.52
*ATG7*	3p25.3	Autophagy related 7	−1.52	−2.13
*LOC387820*	11q24.3	DnaJ (Hsp40) homolog, subfamily B, member 7 pseudogene	1.51	
*PAK1*	11q13-q14	p21 protein (Cdc42/Rac)-activated kinase 1	1.52	
*COMMD1*	2p15	Copper metabolism (Murr1) domain containing 1	1.54	
*RAB11A*	15q22.31	Member RAS oncogene family	1.55	
*EMP3*	19q13.3	Epithelial membrane protein 3	1.76	
*RSAD2*	2p25.2	Radical S-adenosyl methionine domain containing 2	1.83	
*MAP3K11*	11q13.1-q13.3	Mitogen-activated protein kinase kinase kinase 11	1.83	
*GSTM1*	1p13.3	Glutathione S-transferase mu 1	1.95	
*GSTM2*	1p13.3	Glutathione S-transferase mu 2 (muscle)	2.01	
*TNFRSF1A*	12p13.2	Tumor necrosis factor receptor superfamily, member 1A	2.02	

Coefficients below zero correspond to elevated gene expression in optimal responders and coefficients above zero correspond to elevated gene expression in non-optimal responders

Pathway enrichment analysis of the differently expressed genes showed the following molecular networks involved (*p* < 0.05): glutathione metabolism, drug and xenobiotic metabolism, chemical mutagenesis, glycosaminoglycan degradation and MAPK signaling pathway. According to the expression levels we may suggest that these pathways are activated in patients who failed to achieve optimal response to TKI therapy.

From the list of the genes associated with cancers we selected first four with the largest difference in expression levels and analyzed their expression with RT-PCR: *DAZAP2, UBA52, PRR13* and *ATG7*. Expression of these genes was measured in the same samples as whole transcriptome analysis and additional 10 samples from optimal responders were added. All the tested genes demonstrated similar differences in expression levels in optimal vice non-optimal responders as in whole transcriptome analysis (Table 4), however these differences were not significant (ANOVA, *p* = 0.3).

### miRNA analysis

After transcriptome analysis we focused on miRNA expression in patients with different TKI efficacy. We had already 140 patients with the follow-up period more than 6 months treated in three Hospitals in Russia. According to ELN2013 recommendations we selected 6 patients with optimal response and 6 patients with failure of the TKI therapy. Patient ages ranged from 20 to 89 years, with a median of 52.5 years. All 12 patients were treated with 400 mg imatinib. Detailed characteristics of these patients are available in Table 5. All patients with therapy failure had > 10% Ph+ cells at 6 months in bone marrow. Four patients were included in both transcriptome and miRNA analysis (#3, 5, 7, and 12 in Tables 3 and 5).

**Table 5:** Characteristics of patients for miRNA study

Patient	Gender	Age, years	Sokal score	TKI first line, dosage	Efficient TKI
3	Female	57	Low risk	Imatinib, 400 mg	Optimal response
5	Female	68	Intermediate risk	Imatinib, 400 mg	Non-optimal response
7	Male	34	Intermediate risk	Imatinib, 400 mg	Non-optimal response
12	Female	38	Low risk	Imatinib, 400 mg	Optimal response
13	Female	26	Low risk	Imatinib, 400 mg	Optimal response
14	Male	56	Low risk	Imatinib, 400 mg	Optimal response
15	Female	65	Low risk	Imatinib, 400 mg	Optimal response
16	Female	32	High risk	Imatinib, 400 mg	Optimal response
17	Male	44	Low risk	Imatinib, 400 mg	Non-optimal response
18	Female	89	Low risk	Imatinib, 400 mg	Non-optimal response
19	Female	63	Intermediate risk	Imatinib, 400 mg	Non-optimal response
20	Male	49	Intermediate risk	Imatinib, 400 mg	Non-optimal response

ANOVA did not reveal significant difference between responders and failures. However since the number of subjects (samples) was very limited and the number of parameters (genes) was high it was reasonable to pay particular attention to the most significant expression changes of individual miRNAs. We selected miRNAs with individual *p*-value < 0.05–18 genes (Table 6). We analyzed these miRNAs using DIANA tools [28]. This set of programs allows pathway enrichment analysis with genes, interacting with selected miRNAs.

**Table 6:** List of selected miRNAs

Gene	Accession	Coefficient^a^	*p*-value
hsa-miR-720	MIMAT0005954	−12.31	0.04
hsa-miR-1180	MIMAT0005825	−8.33	0.02
hsa-miR-92a-3p	MIMAT0000092	−7.58	0.03
hsa-miR-409-3p	MIMAT0001639	−3.06	0.03
hsa-miR-331-5p	MIMAT0004700	−2.68	0.01
hsa-miR-662	MIMAT0003325	−2.5	0.03
hsa-miR-891a	MIMAT0004902	−2.5	0.04
hsa-miR-548ah-5p	MIMAT0018972	− 2.22	0.01
hsa-miR-432-5p	MIMAT0002814	−2.2	0.03
hsa-miR-553	MIMAT0003216	1.32	0.02
hsa-miR-338-3p	MIMAT0000763	2.91	0.02
hsa-miR-450a-5p	MIMAT0001545	3.29	0.03
hsa-miR-145-5p	MIMAT0000437	3.48	0.01
hsa-miR-324-5p	MIMAT0000761	3.8	0.03
hsa-miR-181b-5p + hsa-miR-181d	MIMAT0000257	4.26	0.03
hsa-miR-181a-5p	MIMAT0000256	4.29	0
hsa-miR-221-3p	MIMAT0000278	5.49	0.02
hsa-miR-4286	MIMAT0016916	26.46	0.01

^a^Coefficients below zero correspond to elevation of miRNA expression in optimal responders and coefficients above zero correspond to elevated miRNA expression in non-optimal responders

We found 40 pathways enriched with genes regulated by the selected miRNAs (Additional file 2: Table S2). These differently expressed miRNAs are involved in vital processes in cells including p53 signaling pathway, toll-like receptor signaling pathway, adherence junction, notch signaling pathway, PI3K-Akt signaling pathway, apoptosis, Wnt signaling pathway, MAPK signaling pathway, transcriptional misregulation in cancer and cell cycle. Among these pathways “Pathways in cancer” (hsa05200) has the highest p-value (9.5*10^− 14^) with 27 genes regulated by 7 miRNAs. Among other cancer specific pathways one can particularly mention “Chronic myeloid leukemia” (hsa05220) (*p* = 0.00018) with 8 genes regulated by 4 miRNAs and “Acute myeloid leukemia” (hsa05221) (*p* = 0.04) with 4 genes regulated by 3 miRNAs.

## Discussion

Here we describe our attempts to find molecular predictors of TKI therapy efficacy in primary CML patients based on genetic variants and expression.

We applied Sokal score to the patients included in transcriptome and miRNA analyses and were surprised by the poor prediction of the TKI efficacy. Seventy-three percent (8 from 11 patients) were correctly predicted as optimal responders and only 56% (5 from 9 patients) were correctly predicted as non-responders. Taking together only 65% of patients in our study were correctly classified based on Sokal score. Sure we clearly understand that our groups are very small for any conclusions about application of Sokal score. But we also understand that it is very difficult to rely on the predictive value of this score for the prediction of early treatment efficacy. At the same time there were no patients with borderline values of BCR-ABL^IS^ or Ph+ metaphases in our transcriptome and miRNA study. All responders really responded well to treatment, and non-responders were > 10% Ph+ cells at 6 months in bone marrow. Therefore, we conducted this study in order to find new molecular predictors of treatment efficacy .

To date, researchers performed several attempts to detect genetic markers associated with resistance to TKI or non-optimal response to targeted therapy. It was shown that the resistance of leukemic cells is associated with the enhanced PI3K/AKT, RAF/MEK/ERK and STAT3 signaling pathways [29]. At the same time, studies involving patients with different responses to TKI, show influence of genetic polymorphisms in TKI metabolizers’ genes [9], *BIM* gene [30], *FAS* gene [10], *DNMT3A* and *ASXL1* genes [11].

We did not confirm different allelic frequencies in patients with different response to the therapy. This can be explained by the fact that the efficiency of TKI therapy can be affected by a variety of both genetic and environmental factors, making the therapy failure a multifactorial feature.

Previously performed studies showed that the expression profiles in patients with optimal and non-optimal responses to TKI therapy varied widely. For example, as published by Frank et al. in 2006, differential expression of some genes was found in two groups of patients and based on this findings authors made conclusions about the discovery of candidate predictors of imatinib resistance [14]. In another study, published in the same year in the same journal, significant difference in the expression of other genes was showed [15]. Finally, one more work in the same journal demonstrated a third list of genes of potential predictors [31]. All these works were done before the next-generation sequencing era, so we conducted our study to find the expression differences by unbiased analysis of the large number of genes. We found some changes in expression levels, but they were very small and it is inappropriate to further look for changes in some specific genes and to develop prognostic tests on their basis. Additionally, we checked all genes that have been previously mentioned in similar studies to be associated with different responses to TKI therapy. We found no difference in allelic frequencies of these genes in our groups. It is possible that revealing specific activation patterns of the whole pathogenic mechanisms is more promising for diagnostic purposes. Another possible solution is to analyze expression of bone marrow samples, containing more stem cells or direct selection and analysis of cancer stem cells which is however difficult for routine implementation.

We revealed 17 differently expressed miRNAs in patients with optimal and non-optimal responses to TKI therapy. For nowadays miRNA profiling is frequently used to find molecular predictors, treatment targets or biomarkers in cancer. Indeed miRNAs are involved in CML pathogenesis [17–19]. Recently it was shown that miR-92a-3p functions as oncogene blocking apoptosis in AML cells. Inhibition of this miRNA leads to increase of apoptosis in M-07e cell line [32]. Nevertheless in our study patients with optimal response had 8-fold higher expression of miR-92a-3p than non-optimal responders. We revealed 3-fold decrease of miR-331-5p in treatment failures. This finding is in the agreement with previously reported association of low levels of this miRNA in K562 cells with the resistance to doxorubicin [33]. We also found that expression of miR-324-5p was 3.8-fold higher in patients with non-optimal response to TKI which correlates with its elevated expression in patients with poor outcome (progression-free and overall survival) of the diffuse large B-cell lymphoma [34]. Despite such large differences in miRNA expression levels between groups, we did not continue this work due to the absence of significant difference using ANOVA with strict corrections for multiple comparisons.

We didn’t sequence *BCR/ABL* kinase domain in all patients, because these mutations usually develop during treatment and result in the secondary resistance to TKIs while we examined patients with primary TKI resistance. Nevertheless we checked 7 out of 11 patients included in transcriptome and miRNA expression analysis and didn’t find mutations in *BCR/ABL* kinase domain (data not shown).

## Conclusions

In conclusion, using modern high-throughput methods such as whole-exome, transcriptome and miRNA analysis, we could not find reliable molecular markers for early prediction of TKI efficiency in Ph+ CML patients. Apparently, efficacy of targeted therapy is determined not only by genetic factors, the environmental component of therapy response also plays a great role. It is necessary to continue search for mechanisms of tumor cells sensitivity to therapy for their complete elimination.

## Additional files

Additional file 1: **Table S1.** List of primers. (XLSX 10 kb)

Additional file 2: **Table S2.** Pathways enriched with genes regulated by the selected miRNAs. (XLSX 11 kb)
